# Genomic Variants and Worldwide Epidemiology of Breast Cancer: A Genome-Wide Association Studies Correlation Analysis

**DOI:** 10.3390/genes15020145

**Published:** 2024-01-23

**Authors:** Giovanna Gilioli da Costa Nunes, Lilian Marques de Freitas, Natasha Monte, Laura Patrícia Albarello Gellen, Aline Pasquini Santos, Francisco Cezar Aquino de Moraes, Ana Caroline Alves da Costa, Milena Cardoso de Lima, Marianne Rodrigues Fernandes, Sidney Emanuel Batista dos Santos, Ney Pereira Carneiro dos Santos

**Affiliations:** 1 Research Center of Oncology, Federal University of Pará Belém, Belém 66073-000, Brazil; 2 Laboratory of Human and Medical Genetics, Institute of Biological Science, Federal University of Pará, Belém 66075-110, Brazil

**Keywords:** breast cancer, genetic risk variants, susceptibility, severity, epidemiology, population

## Abstract

Breast cancer (BCa) is the most common cancer and leading cause of cancer death among women globally. This can be explained by the genetic factor of this disease. This article aims to correlate the epidemiological data, worldwide incidence, and mortality of BCa with the Single-Nucleotide Polymorphisms (SNPs) associated with the susceptibility and severity in different populations. Two hundred and forty genetic variants associated with BCa susceptibility/severity were selected from the literature through Genome-Wide Association Studies (GWAS). The allele frequencies were obtained from the 1000 Genomes Project, and the epidemiological data were obtained from the World Health Organization (WHO). The BCa incidence, mortality rates, and allele frequencies of the variants were evaluated using Pearson’s correlation. Our study demonstrated that 11 SNPs (rs3817578, rs4843437, rs3754934, rs61764370, rs780092, rs2290203, rs10411161, rs6001930, rs16886165, rs8051542 and rs4973768) were significantly correlated with the epidemiological data in different ethnic groups. Seven polymorphisms (rs3817578, rs3754934, rs780092, rs2290203, rs10411161, rs6001930 and rs16886165) were inversely correlated with the incidence rate and four polymorphisms (rs4843437, rs61764370, rs8051542 and rs4973768) were directly correlated with the incidence rate. African and South-East Asian populations have a lower risk of developing BCa when evaluated in terms of genetic factors since they possess variants characterized as protective, as their higher incidence is associated with a lower frequency of BCa cases. The genetic variants investigated here are likely to predispose individuals to BCa. The genetic study described here is promising for implementing personalized strategies to screen for breast cancer in diverse populations.

## 1. Introduction

Female breast cancer (BCa) is the fifth leading cause of cancer mortality worldwide and surpassed lung cancer as the leading cause of the global cancer incidence in 2020, representing 11.7% of all cancer cases, and it is expected to represent 15.2% of all new cancer cases in 2023 [1,2,3].

The etiology of BCa is a multifactorial complex determined both by genetic and non-genetic factors that include advanced age, positive family history, and Caucasian ancestry and is highly correlated with human development. The elevated incidence rates observed in regions characterized by higher Human Development Index (HDI) levels correlate with the persistent prevalence of reproductive and hormonal risk factors. These factors encompass precocious onset of menarche, delayed menopausal onset, advanced maternal age at primiparity, reduced parity, diminished instances of breastfeeding, utilization of menopausal hormone therapies, and oral contraceptive use. Concurrently, lifestyle-related risks, notably alcohol consumption, excessive adiposity, and physical inactivity, contribute significantly. Additionally, the augmented detection rates are attributable to structured or opportunistic mammographic screening programs [4].

The BCa incidence varies across ethnic groups and geographic variations. There are higher incidence and mortality rates in people of Caucasian/Non-Hispanic White descent worldwide. Twin investigations within Nordic societies have revealed heritability estimates reaching 31%, while GWAS conducted on individuals of Caucasian descent, including those of American and European origin, have unveiled heritability estimates up to 13%. Contrastingly, mortality rates within African regions have seen a concerning surge, positioning them among the highest globally. The diminished survival rates witnessed in sub-Saharan Africa predominantly stem from delayed-stage disease presentations [3,5]. 

Pathogenic variants found in BRCA1/BRCA2 and certain variations known as SNPs are widely recognized as significant factors in explaining a substantial portion of the breast cancer risk among women with a strong family history of the disease. These, along with mutations in genes like ATM, BARD1, PALB2, and CHECK2, which have an intermediate impact, collectively account for up to 25% of the risk of developing breast cancer. However, a significant portion of the genetic tendency remains unexplained, likely due to low-impact variants. SNPs also play a role in the development of breast cancer in women without familial ties to the disease, contributing to approximately 16% of the genetic risk. Looking at the entire population, the combined risk arising from these susceptibility SNPs surpasses the risk posed by individual harmful mutations in a single high- or moderate-risk gene, particularly among women without any family history of breast cancer [6,7].

Many studies have shown that different molecular subtypes of BCa have different genetic variables, which may indicate that different subtypes of BCa have different etiological pathways. With the development of GWAS, an increasing number of genetic variables have been confirmed to be associated with BCa [8]. 

GWAS, or Genome-Wide Association Studies, is a technology tool utilized to sift through vast numbers of single-nucleotide polymorphisms and other genetic variations within human genomes. This technique, conducted using microarray or sequencing methods, aims to identify specific gene locations associated with various diseases [9]. Identifying numerous risk loci aids in enhancing our understanding of disease mechanisms and prognosis, and it substantiates the multifaceted genetic foundation underlying breast cancer. Collectively, these investigations significantly contribute to our comprehension of the development and progression of breast cancer.

This article aims to correlate epidemiological data on the incidence and mortality of BCa worldwide with the frequencies of important SNPs in studies of GWAS associated with the susceptibility and severity of this neoplasm in different populations.

## 2. Materials and Methods

### 2.1. SNP Determination

**Figure 1:**
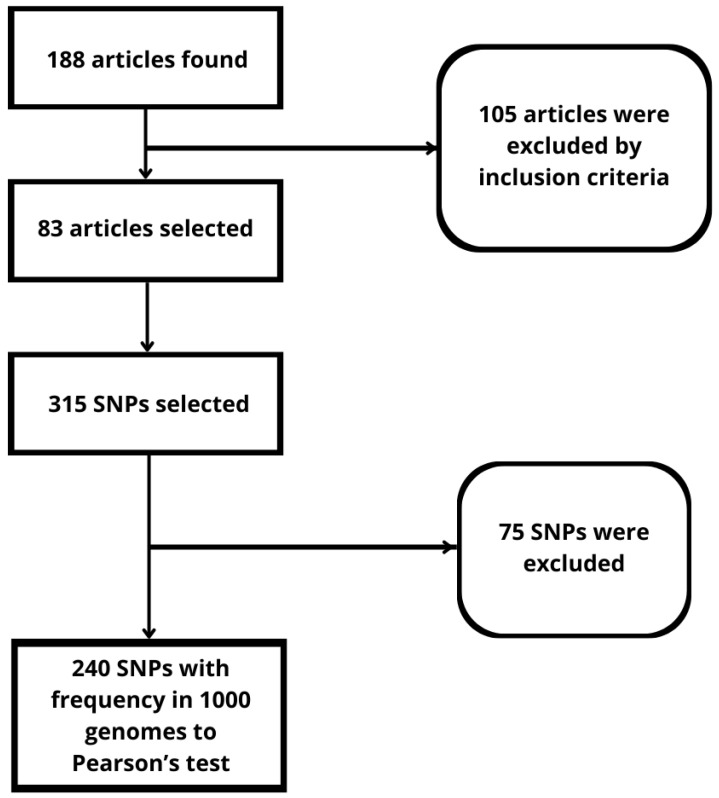
Methodology for selection of the genetic variants.

The data extraction process was conducted independently by three researchers (G.G.d.C.N., L.M.d.F. and A.P.S.). We searched the online Medline/PubMed databases for articles published in English using several key terms related to breast cancer. The key terms were “breast cancer”, “GWAS”, and “risk OR susceptibility OR mortality”. The filters used were published between January 2017 and July 2022. There were 188 articles (Figure 1) after using the following inclusion criteria: studies on SNPs associated with risk and/or susceptibility and/or mortality concerning breast cancer.

We included GWAS, cohort, case-control and meta-analysis studies on the association of the SNP with the BC incidence or mortality. We excluded observational studies without a control group, case reports, case series, literature reviews, editorials and book chapters.

### 2.2. Epidemiological and Genetic Data

The incidence and mortality rates of breast cancer for the population were obtained from the World Health Organization (WHO) platform, available on the Global Cancer Observatory [10]. 

In the 1000 Genomes Project, the allele frequencies of genetic variants in continental populations were evaluated: Europe (EUR), Africa (AFR), East Asia (EAS), South Asia (SAS) and Americas (AMR). We compared the WHO populations with the continental populations available in phase 3 of the 1000 Genomes URL https://www.internationalgenome.org/ (accessed on 2 January 2024) database. According to the Consortium of the 1000 Genomes Project, populations are grouped by the predominant component of their ancestry, so our study correlated the epidemiological and genetic data of the populations of EUR, AFR, EAS, SAS and AMR. For the statistical analyses, the East and South Asian populations were grouped due to their genetic similarities.

### 2.3. Statistical Analysis

The Pearson’s correlation was used for the BCa incidence, mortality rates, and allele frequencies of the variants. The data were evaluated with previously described groups, using the “cor. test” function of the “stats” package of the R programming language. Thereafter, and after passing Bonferroni correction *p*-value threshold, the values of the r, r2, *p*-value, and 95% CI were obtained. All the plots were created using the “ggplot2” graphics package. It considered only *p*-value less than 0.05 (*p* ≤ 0.05) as statistically significant.

## 3. Results

The Age-Standardized Incidence Rate (ASIR) for breast cancer indicates the frequency of new diagnoses of breast cancer within a defined timeframe, considering the distribution of age in the population. The Age-Standardized Mortality Rate (ASMR) for breast cancer is calculated based on the count of deaths caused by the disease within a specific period, adjusting for the age distribution of the population.

**Figure 2:**
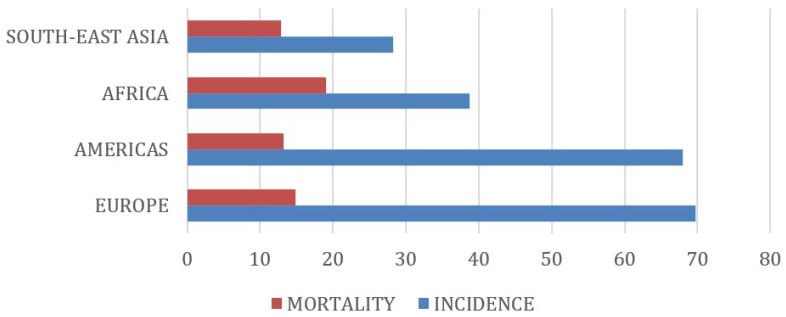
Incidence and mortality rates per 100,000 habitants from the World Health Organization International Agency for Research on Cancer in different populations.

The incidence rate (ASIR) of BCa was 69.7 per 100,000 females in the European population, followed by the American (68), African (38.7) and South-East Asian populations (28.3). On the other hand, the mortality rate (ASMR) of BCa was higher in the African population at 19.1 per 100,000, followed by the European (14.8), American (13.2) and South-East Asian populations (12.9) (Figure 2).

**Figure 3:**
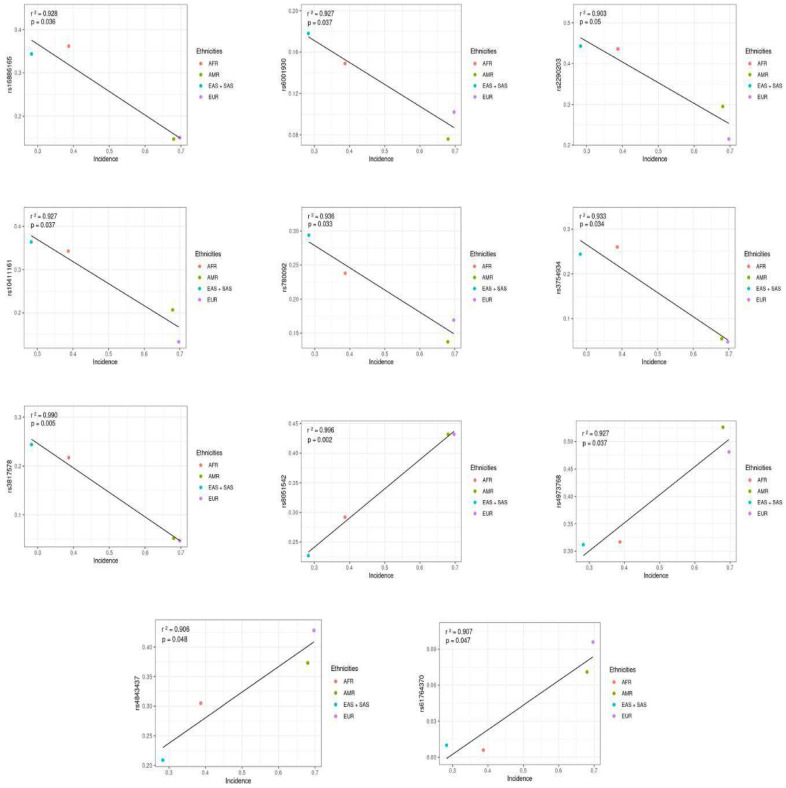
SNPs associated with the BCa incidence in different populations.

A total of 240 SNPS (Supplementary Table S1) were selected from the literature, as described in Figure 1. Among those polymorphisms, 11 SNPs (rs3817578, rs4843437, rs3754934, rs61764370, rs780092, rs2290203, rs10411161, rs6001930, rs16886165, rs8051542 and rs4973768) were significant according to the Pearson’s correlation analysis (Table 1). All of the 11 significant SNPs were positively correlated only with the incidence rate (Figure 3), once the SNPs correlated with the mortality rate and toxicity events were less significant (p value > 0.05).

Analyzing the correlation analyses between the variants and the epidemiological data of each specific population group, a recurring pattern becomes evident across all the correlations. In the directly related analyses where the higher frequency of the variant corresponded to a greater incidence of BCa, the European and American populations were at the positive end (with a higher frequency of the variant and higher incidence), while the other populations, African and South-East Asian, were at the opposite end (lower frequency of the variant and lower BCa incidence).

Conversely, in the inversely related analyses where the higher frequency of the variant corresponded to a lower BCa incidence, the African and South-East Asian populations were at the positive end (higher frequency of the variant and lower incidence), while the other populations, European and American, were at the opposite end (lower frequency of the variant and higher BCa incidence). Thus, we can conclude that African and South-East Asian populations have a lower risk of developing BCa when evaluated in terms of genetic factors since they possess variants characterized as protective, as their higher incidence is associated with a lower frequency of BCa cases.

Of all the eleven variants (rs3817578, rs4843437, rs3754934, rs61764370, rs780092, rs2290203, rs10411161, rs6001930, rs16886165, rs8051542 and rs4973768), four of them (rs4843437, rs61764370, rs8051542 and rs4973768) were directly correlated with the incidence rate of BCa. The higher the frequency of the variant, the greater the number of new cases of BCa. Eventually, these polymorphisms were more frequent in the European population, followed by the American population, which indicates high susceptibility to BCa in these populations.

The variants rs4843437 and rs8051542 were described as directly correlated in the analysis and demonstrated that the higher the frequency of the variant, the higher the incidence of that variant in certain populations. Both revealed a higher frequency in the European population, followed by the American population, with these being the populations with the highest incidences of BCa in the world. On the other hand, we noticed a lower frequency of these variants in the South-East Asian population, followed by the African population, with these being the populations with the lowest incidences of BCa.

The directly correlated rs4973768 variant presented a lower frequency of the variant and a lower incidence of BCa in the South-East Asian population, followed by the African population, and presented a higher frequency in the American population and a higher incidence in the European population.

The rs61764370 variant, also directly correlated in the analysis, showed a lower frequency of the variant and a lower incidence of BCa in the South-East Asian population, followed by the African population. However, unlike the rs4973768 variant, the variant had a higher frequency and higher incidence in the European population, followed by the American population.

Nevertheless, seven polymorphisms (rs3817578, rs3754934, rs780092, rs2290203, rs10411161, rs6001930 and rs16886165) were inversely correlated with the incidence rate. Consequently, the higher the frequency of the variant allele, the lower the estimated incidence rate. All of the seven variants were more frequent in the South-East Asian population, possibly because South-East Asian ancestry may be a protective factor against BCa.

When we analyze the inversely and the directly correlated variants, we can observe the trend already described, albeit with some particularities. The rs16886165 and the rs3754934 variants have a significantly higher frequency in the African and South-East Asian populations and an oppositely lower BCa incidence in these populations, as we can also see from the analysis of the epidemiological data (Figure 2). Despite the higher frequency in the African population, the incidence of BCa remains lower in South-East Asian population. Both reveal a higher incidence of BCa in the European population; however, while the rs16886165 variant is more common in the European population, the rs3754934 variant is more common in the American population.

The rs6001930 and the rs780092 variants, also inversely correlated, demonstrated a clearer relationship between a higher frequency of the variant and a lower incidence of the variant in the South-East Asian population, revealing a clear agreement between the epidemiological data of the Asian population (Figure 2) and the genetic factors. However, among the American and European populations, those with the highest incidence rates of BCa, the European population showed a higher frequency of the rs6001930 and the rs780092 variants when compared to the American population.

Analysis of the variants rs2290203, rs10411161 and rs3817578 also revealed an inversely correlated trend between the variant frequency and BCa incidence among populations. Furthermore, we noticed a complete agreement between the epidemiological data (Figure 2) and the genetic risk factors, as the variants were more frequent in the South-East Asian population, followed by the African population, and a lower incidence of BCa in the Asian population, followed by the African population. At the other extreme of the analysis, while the European population presented a lower frequency of these variants, followed by the American population, these populations presented the highest incidences of BCa among the populations analyzed, with the highest incidence in the European population.

The rs16886165 variant has a significantly higher frequency in the African and South-East Asian populations and an oppositely lower BCa incidence in these populations, as we can also see from the analysis of the epidemiological data (Figure 2). Despite the higher frequency in the African population, the incidence of BCa remains lower in South-East Asian population.

## 4. Discussion

The BCa incidence rates vary significantly by ethnicity, results that show a wide fluctuation in the epidemiological rates and evidence a higher incidence in females of European descent and worse prognosis in females of African descent. Our results showed that populations of European and American origin had higher incidence rates of the disease, and, contrary to populations of South-East Asian origin, they obtained better results related to susceptibility.

This study correlated epidemiological data on the incidence of BCa worldwide with the frequencies of important SNPs in GWAS associated with the susceptibility of this neoplasm in different populations. Our results indicated correlations for 11 genetic variants (rs3817578, rs4843437, rs3754934, rs61764370, rs780092, rs2290203, rs10411161, rs6001930, rs16886165, rs8051542 and rs4973768) in 10 genes (*CASP8*, *LINC02188*, *KRAS*, *GCKR*, *IQGAP1*, *ZNF577*, *MRTFA*, *RMEL3*, *SLC4A7* and *TOX3*) with the incidence rate. Our results show that the variants related to a high incidence are more frequent in the European and American populations and less frequent in the South-East Asian population.

Cysteine apoptosis-related peptidase (*CASP8*) is a gene involved in programmed cell death. Our analysis showed a significant correlation between the rs3754934 and rs3817578 polymorphisms. Both variants were inversely correlated with the incidence rate and were more frequent in the Southeast Asian population. Our findings corroborate international studies such as the meta-analysis carried out by Hashemi et al. (2020), which also found their association with a reduced risk of cancer, including breast neoplasms [11]. CASP8 serves as a key trigger for both the extrinsic and intrinsic apoptotic pathways upon activation by the Fas cell surface death receptor (FAS) and Fas-associated death domain (FADD). Once activated, CASP8 can function independently or in conjunction with other initiator CASPs. This collaboration results in the activation of downstream effector CASPs, such as CASP3, culminating in the culmination of the apoptotic pathway.

The *LINCO2188* gene is an intergenic RNA not coding for the long protein 2188. In our study, rs4843437 was associated with a higher incidence, being more frequent in Europeans and Americans. In contrast, Qingxia et al. (2023) found an association with increased immune infiltration of tumor tissue, activating various immune cells in the basal subtype of breast cancer and inhibiting tumor development [12]. 

KRAS is an important predictive pharmacogene of cancer and is one of the most frequent gain-of-function alterations found in patients with cancer. Oncogenic mutations in KRAS trigger persistent activation of KRAS-dependent pathways and have been detected across various cancer types. It is estimated that around 25% of all cancer cases involve, at least in part, a KRAS mutation. In our investigations, the rs61764370 variant was linked to a heightened incidence and was more prevalent among European and African populations. While there is limited literature associating this gene with breast cancer, recent studies, such as that conducted by Zeng et al. in 2023, have shed light on the correlation. Despite KRAS mutations being more commonly observed in colorectal cancer, these studies revealed a noteworthy connection between the onset of abnormal tissue growth and the formation of distant metastases, promoting the development of secondary tumors [13].

GCKR operates as a crucial regulatory protein for glucokinase (GCK) in the β cells of the liver and pancreas. Variations in this gene have shown robust associations with diabetes. In our investigation, the genetic variant (rs780092) within this gene exhibited an inverse correlation with breast cancer occurrence [14,15]. IQGAP1, a multifaceted scaffold protein, orchestrates various cellular functions encompassing cell adhesion, transcriptional regulation, cytoskeletal arrangement, and signaling pathways. The existing literature has suggested the potential involvement of IQGAP1 in cancer by modulating signaling pathways pivotal in cell proliferation and transformation [16]. In our study, the variant rs2290203 demonstrated an inverse relationship with the breast cancer incidence rates, notably prevalent in the South-East Asian population, indicating a potential protective aspect within this group. ZNF577, a gene involved in encoding a protein with DNA-binding transcription factor activity, has been associated with altered levels of methylation in leukocytes, particularly linked to obesity [17]. In addition, recent studies have revealed an association between *ZNF577* methylation levels and breast cancer, pointing to this gene as a potential biomarker of the effect of environmental factors on breast cancer. In our analysis, we found an association with the breast cancer incidence rates in a variant (rs10411161) present in this gene. 

Myocardin-related transcription factor A (*MRTFA*) is the coactivator of the serum response factor (SRF) [18] transcription factor, which induces the transcription of genes involved in cell migration, adhesion, structure, growth and apoptosis [19,20]. *MRTFA* plays an important role in the interaction pathways in breast cancer [21]. In our study, the SNP in *MRTFA* (rs6001930) represents a lower incidence rate, being more frequent in the Southeast Asian population.

Pioneering studies on *RMEL3* indicated higher survival rates without gene expression [22]. *RMEL3* is involved in protein kinase A signaling, molecular mechanisms of cancer, regulation of epithelial-mesenchymal transition, matrix metalloprotease protection, in addition to the ability to promote malignancy [23]. More recent research has discovered that the gene acts in cellular support, conservation and tumor growth [24]. The results of this study indicated that the variant in *RMEL3* (rs16886165) is inversely proportional to the BCa incidence rates and was more frequent in the Southeast Asian population.

*SLC4A7* has potential as a tyrosine kinase substrate, encodes the sodium bicarbonate transporter, and also affects the intracellular pH in breast cancer. The presence of the rs4973768 gene variant directly implies susceptibility to BCa and is responsible for ~0.4% of the familial risk of BCa [25,26]. The results of this study also found a direct relationship between the presence of the *SLC4A7* rs4973768 variant and higher incidence rates of BCa, being more present in the European population.

*TOX3* interacts with the estrogen response pathways in the neural system, acts on cell invasion, metastasis, downregulation of *BRCA1*, proliferation of ovarian granulosa cells and inhibits cell apoptosis [27]. In some studies, the SNPs in the gene have been associated with a worse prognosis and increased risk of BCa [8]. In our study, the SNP in *TOX3* (rs8051542) was directly correlated with the incidence rate of BCa and was found to be more frequent in the European population.

## 5. Conclusions

Breast cancer is the most common cancer and leading cause of cancer death among women globally. GWAS studies have increasingly clarified the main genetic targets related to the possible predisposition to breast cancer. Therefore, due to its complexity and heterogeneity, BCa has many potentially targetable mutations involved in cancer progression. Cancer precision medicine needs to be deeply and widely explored through studies to achieve the goal of individualized treatment and improved patient prognosis. Our study carried out an analysis correlating different GWAS studies and demonstrated that 11 SNPs (rs3817578, rs4843437, rs3754934, rs61764370, rs780092, rs2290203, rs10411161, rs6001930, rs16886165, rs8051542 and rs4973768) were all significantly related to the incidence rate of breast cancer in different ethnic groups. Identifying numerous risk loci aids in enhancing our understanding of the disease mechanisms and prognosis, and it substantiates the multifaceted genetic foundation underlying breast cancer.

## Figures and Tables

**Table 1:** SNPs significantly correlated with the BCa incidence in different populations.

Gene	SNP ID/Association	Most Severe Consequence	Alleles	Ancestral	*p* Value	Frequencies			
						AFR	AMR	EUR	SAS + EAS
CASP8	rs3817578/ Incidence	Intron Variant	C/G/T	C	0.005	0.217	0.052	0.047	0.244
LINC02188	rs4843437/ Incidence	Splice Polypyrimidine Tract Variant	T/A/C	T	0.048	0.305	0.373	0.428	0.208
CASP8	rs3754934/ Incidence	Intron Variant	C/A/T	C	0.034	0.260	0.055	0.048	0.244
KRAS	rs61764370/ Incidence	3 Prime UTR Variant	A/C	A	0.047	0.006	0.071	0.096	SAS only (0.020)
GCKR	rs780092/ Incidence	Intron Variant	A/G/T	A	0.033	0.238	0.137	0.169	0.293
IQGAP1	rs2290203/ Incidence	3 Prime UTR Variant	G/A	A	0.05	0.436	0.295	0.215	0.442
ZNF577	rs10411161/ Incidence	3 Prime UTR Variant	C/T	C	0.037	0.343	0.207	0.133	0.364
MRTFA	rs6001930/ Incidence	Intron Variant	T/C/G	C	0.037	0.149	0.076	0.102	0.178
RMEL3	rs16886165/ Incidence	Intergenic Variant	T/C/G	G	0.036	0.362	0.147	0.150	0.343
SLC4AZ	rs4973768/ Incidence	3 Prime UTR Variant	C/T	T	0.037	0.317	0.526	0.481	0.312
TOX3	rs8051542/ Incidence	Intron Variant	T/C/G	C	0.002	0.292	0.432	0.432	0.226

## Data Availability

All relevant data will be shared as Supporting Information files if the manuscript is accepted for publication.

## Supplementary Materials

The following supporting information can be downloaded at https://www.mdpi.com/article/10.3390/genes15020145/s1, Table S1: SNPs correlated with the BCa incidence, mortality and toxicity in different populations.
