# Supplementary material for: Genomic Variants and Worldwide Epidemiology of Breast Cancer: A Genome-Wide Association Studies Correlation Analysis
Source: Genes (Basel). 2024 Jan 23;15(2):145. doi: 10.3390/genes15020145 (PMC10888129; doi:10.3390/genes15020145)
Supplement: Supplementary file 1 [file genes-15-00145-s001.zip › genes-2776110-supplementary.pdf]

Supplementary Table S1. SNPs correlated with PCa incidence, mortality and toxicity in different populations

rs	CATEGORY	Freq AFR	Freq AMR	Freq EAS	Freq EUR	Freq SAS
rs13387042	RISK	0.778	0.363	0.097	0.520	0.484
rs4973768	RISK	0.317	0.526	0.185	0.481	0.439
rs4844616	RISK	0.268	0.512	0.589	0.597	0.374
rs1219648	RISK	0.438	0.406	0.382	0.431	0.376
rs2981579	RISK	0.661	0.427	0.449	0.451	0.389
rs11200014	RISK	0.190	0.376	0.289	0.443	0.326
rs7696175	RISK	0.018	0.343	0.152	0.427	0.247
rs13281615	RISK	0.459	0.566	0.514	0.443	0.507
rs16886165	RISK	0.362	0.147	0.340	0.150	0.347
rs1801320	RISK	0.229	0.085	0.152	0.077	0.128
rs2363956	RISK	0.505	0.390	0.312	0.573	0.490
rs10941679	RISK	0.182	0.386	0.487	0.233	0.357
rs6504950	RISK	0.359	0.177	0.101	0.269	0.188
rs999737	RISK	0.008	0.166	0.003	0.210	0.088
rs1011970	RISK	0.344	0.389	0.105	0.155	0.257
rs2380205	RISK	0.644	0.298	0.130	0.443	0.248
rs10995190	RISK	0.174	0.111	0.022	0.151	0.078
rs704010	RISK	0.030	0.388	0.309	0.413	0.311
rs10093411	RISK	0.671	0.537	0.607	0.246	0.180

rs61229336	RISK	0.098	0.205	0.175	0.332	0.274
rs62521280	RISK	0.008	0.049	0.001	0.107	0.086
rs2981578	RISK	0.078	0.488	0.436	0.481	0.509
rs4784227	RISK	0.042	0.313	0.255	0.254	0.222
rs67397200	RISK	0.275	0.163	0.002	0.262	0.115
rs4245739	RISK	0.231	0.285	0.050	0.260	0.262
rs3757322	RISK	0.501	0.251	0.336	0.298	0.301
rs9397437	RISK	0.031	0.048	0.312	0.072	0.061
rs2747652	RISK	0.490	0.473	0.423	0.447	0.480
rs6678914	RISK	0.314	0.293	0.269	0.431	0.285
rs4577244	RISK	0.197	0.452	0.790	0.227	0.301
rs7072776	RISK	0.563	0.346	0.055	0.286	0.208
rs62355902	RISK	0.090	0.225	0.370	0.149	0.334
rs10069690	RISK	0.662	0.220	0.169	0.276	0.271
rs2981579	RISK	0.661	0.427	0.449	0.451	0.389
rs3817198	RISK	0.137	0.182	0.103	0.314	0.360
rs8170	RISK	0.186	0.095	0.001	0.163	0.088
rs5750715	RISK	0.163	0.357	0.471	0.274	0.371
rs12628403	RISK	0.008	0.167	0.341	0.072	0.156
rs2981575	RISK	0.633	0.428	0.429	0.435	0.390

rs865686	RISK	0.507	0.317	0.080	0.370	0.122
rs9485372	RISK	0.205	0.192	0.423	0.164	0.216
rs2981582	RISK	0.495	0.418	0.316	0.420	0.344
rs2420946	RISK	0.558	0.427	0.384	0.424	0.385
rs12443621	RISK	0.470	0.510	0.586	0.481	0.415
rs8049226	RISK	0.247	0.491	0.188	0.598	0.388
rs3112625	RISK	0.444	0.408	0.202	0.567	0.544
rs726501	RISK	0.002	0.144	0.303	0.092	0.297
rs7120258	RISK	0.161	0.065	0.116	0.106	0.126
rs498337	RISK	0.144	0.352	0.534	0.295	0.267
rs2107425	RISK	0.554	0.474	0.398	0.304	0.486
rs13387042	RISK	0.778	0.363	0.097	0.520	0.484
rs3757318	RISK	0.023	0.053	0.258	0.080	0.044
rs1926657	RISK	0.324	0.223	0.307	0.185	0.307
rs6556756	RISK	0.045	0.095	0.403	0.111	0.134
rs3817198	RISK	0.137	0.182	0.103	0.314	0.360
rs8051542	RISK	0.292	0.432	0.178	0.432	0.275
rs17468277	RISK	0.043	0.075	0.001	0.120	0.024
rs16886165	RISK	0.362	0.147	0.340	0.150	0.347
rs1154865	RISK	0.217	0.304	0.135	0.240	0.167

rs12922061	RISK	0.045	0.314	0.269	0.232	0.222
rs2290203	RISK	0.436	0.295	0.513	0.215	0.372
rs1045485	RISK	0.050	0.076	0.001	0.120	0.024
rs13431652	RISK	0.145	0.184	0.050	0.311	0.163
rs573225	RISK	0.031	0.202	0.057	0.323	0.165
rs560887	RISK	0.011	0.187	0.022	0.299	0.101
rs13182814	RISK	0.445	0.222	0.079	0.262	0.263
rs35009176	RISK	0.445	0.222	0.079	0.262	0.263
rs34411024	RISK	0.445	0.222	0.079	0.262	0.263
rs6555516	RISK	0.443	0.222	0.080	0.262	0.263
rs6555517	RISK	0.443	0.222	0.079	0.262	0.263
rs6555518	RISK	0.247	0.202	0.079	0.259	0.257
rs7447098	RISK	0.247	0.202	0.079	0.259	0.256
rs6555519	RISK	0.443	0.222	0.081	0.261	0.260
rs7447152	RISK	0.247	0.202	0.081	0.258	0.257
rs7444691	RISK	0.247	0.202	0.080	0.258	0.257
rs17131	RISK	0.443	0.220	0.080	0.262	0.262
rs13169903	RISK	0.247	0.202	0.079	0.259	0.260
rs1847915	RISK	0.445	0.222	0.079	0.262	0.263
rs6555520	RISK	0.446	0.222	0.079	0.262	0.263

rs6555521	RISK	0.248	0.202	0.079	0.259	0.260
rs2123640	RISK	0.097	0.305	0.094	0.261	0.256
rs7716902	RISK	0.067	0.300	0.094	0.260	0.256
rs13188458	RISK	0.096	0.305	0.094	0.260	0.258
rs13188952	RISK	0.096	0.305	0.094	0.260	0.258
rs10512942	RISK	0.061	0.300	0.094	0.259	0.258
rs34799743	RISK	0.067	0.298	0.094	0.260	0.259
rs13166872	RISK	0.067	0.298	0.094	0.259	0.258
rs17198862	RISK	0.090	0.298	0.095	0.262	0.264
rs1432679	RISK	0.147	0.389	0.389	0.553	0.664
rs10759243	RISK	0.674	0.375	0.450	0.308	0.401
rs10822013	RISK	0.178	0.523	0.505	0.504	0.505
rs621414	RISK	0.045	0.519	0.481	0.567	0.484
rs887953	RISK	0.474	0.288	0.138	0.348	0.293
rs7641929	RISK	0.675	0.334	0.157	0.385	0.297
rs2548998	RISK	0.278	0.390	0.293	0.534	0.505
rs527078	RISK	0.741	0.256	0.048	0.335	0.182
rs680096	RISK	0.384	0.320	0.219	0.377	0.396
rs8028277	RISK	0.582	0.254	0.444	0.287	0.397
rs4545784	RISK	0.518	0.238	0.344	0.286	0.415

rs4886708	RISK	0.574	0.254	0.448	0.282	0.399
rs6542583	RISK	0.220	0.411	0.596	0.291	0.385
rs2041692	RISK	0.176	0.235	0.512	0.206	0.607
rs9383932	RISK	0.150	0.098	0.386	0.131	0.160
rs9397435	RISK	0.072	0.049	0.312	0.074	0.060
rs12662670	RISK	0.039	0.063	0.306	0.081	0.050
rs12665607	RISK	0.016	0.061	0.313	0.080	0.061
rs9383589	RISK	0.022	0.062	0.313	0.079	0.046
rs3734805	RISK	0.031	0.062	0.315	0.079	0.046
rs6929137	RISK	0.496	0.245	0.333	0.295	0.298
rs7752591	RISK	0.261	0.625	0.566	0.495	0.516
rs3734804	RISK	0.244	0.620	0.572	0.496	0.530
rs6932260	RISK	0.243	0.620	0.570	0.496	0.528
rs852003	RISK	0.260	0.575	0.567	0.428	0.434
rs2046210	RISK	0.663	0.272	0.362	0.321	0.318
rs2981582	RISK	0.495	0.418	0.316	0.420	0.344
rs889312	RISK	0.337	0.411	0.535	0.283	0.392
rs3803662	RISK	0.567	0.390	0.619	0.291	0.273
rs10069690	RISK	0.662	0.220	0.169	0.276	0.271
rs8170	RISK	0.186	0.095	0.001	0.163	0.088

rs10771399	RISK	0,034	0,072	0,177	0,106	0,138
rs1292011	RISK	0,446	0,375	0,234	0,414	0,621
rs9383935	RISK	0,03	0,062	0,314	0,079	0,045
rs2228480	RISK	0,145	0,238	0,201	0,175	0,214
rs3798758	RISK	0,136	0,124	0,258	0,031	0,123
rs1323697	RISK	0,447	0,248	0,382	0,184	0,188
rs1264308	RISK	0,024	0,022	0,004	0,08	0,042
rs1469412	RISK	0,216	0,242	0,14	0,214	0,226
rs1092913	RISK	0,322	0,357	0,687	0,097	0,342
rs10411161	RISK	0,343	0,207	0,297	0,133	0,431
rs3848562	RISK	0,482	0,225	0,299	0,133	0,427
rs1429142	RISK	0.790	0.255	0.328	0.159	0.388
rs1981867	RISK	0.132	0.295	0.387	0.311	0.420
rs1864182	RISK	0.821	0.389	0.092	0.446	0.303
rs10514231	RISK	0.697	0.294	0.101	0.353	0.259
rs616488	RISK	0.086	0.496	0.317	0.326	0.242
rs17530068	RISK	0.036	0.239	0.247	0.235	0.184
rs6001930	RISK	0.149	0.076	0.240	0.102	0.116
rs1830298	RISK	0.189	0.432	0.305	0.283	0.138
rs10197246	RISK	0.180	0.442	0.294	0.290	0.134

rs8176318	RISK	0.157	0.284	0.372	0.357	0.498
rs120963	RISK	0.483	0.274	0.303	0.187	0.313
rs6707272	RISK	0.379	0.244	0.258	0.300	0.302
rs13014061	RISK	0.037	0.245	0.251	0.343	0.303
rs560304	RISK	0.486	0.285	0.255	0.344	0.304
rs1942574	RISK	0.007	0.137	0.061	0.160	0.155
rs7895676	RISK	0.082	0.493	0.428	0.467	0.496
rs4415084	RISK	0.350	0.442	0.453	0.591	0.515
rs6451770	RISK	0.441	0.539	0.548	0.389	0.447
rs12515012	RISK	0.353	0.484	0.492	0.403	0.485
rs13156930	RISK	0.368	0.441	0.453	0.594	0.510
rs16901937	RISK	0.313	0.490	0.492	0.406	0.484
rs851980	RISK	0.225	0.242	0.097	0.250	0.150
rs3778609	RISK	0.381	0.219	0.302	0.020	0.209
rs12918816	RISK	0.399	0.366	0.474	0.280	0.223
rs2912780	RISK	0.684	0.429	0.450	0.452	0.389
rs45631563	RISK	0.005	0.035	0.002	0.057	0.069
rs17817449	RISK	0.376	0.249	0.170	0.415	0.289
rs13329835	RISK	0.755	0.195	0.039	0.242	0.067
rs941764	RISK	0.781	0.448	0.137	0.353	0.270

rs17529111	RISK	0.052	0.245	0.218	0.223	0.190
rs17529111	RISK	0.052	0.245	0.218	0.223	0.190
rs1053338	RISK	0.012	0.192	0.148	0.147	0.131
rs6964587	RISK	0.502	0.357	0.162	0.387	0.409
rs10510592	RISK	0.120	0.353	0.100	0.274	0.284
rs16857609	RISK	0.232	0.340	0.582	0.274	0.573
rs2420946	RISK	0.558	0.427	0.384	0.424	0.385
rs16917302	RISK	0.394	0.125	0.187	0.116	0.085
rs17221319	RISK	0.075	0.481	0.569	0.491	0.459
rs9348512	RISK	0.352	0.399	0.187	0.323	0.577
rs6975524	RISK	0.805	0.311	0.358	0.335	0.255
rs184577	RISK	0.137	0.163	0.014	0.207	0.097
rs4442975	RISK	0.694	0.354	0.101	0.509	0.483
rs2000999	RISK	0.054	0.173	0.310	0.192	0.433
rs12150660	RISK	0.040	0.141	0.001	0.242	0.070
rs13397985	RISK	0.104	0.114	0.001	0.169	0.149
rs780094	RISK	0.132	0.360	0.476	0.411	0.198
rs11229030	RISK	0.673	0.278	0.159	0.381	0.322
rs780092	RISK	0.238	0.137	0.338	0.169	0.249
rs4788815	RISK	0.216	0.281	0.213	0.364	0.449

rs2571391	RISK	0.270	0.401	0.299	0.357	0.226
rs498872	RISK	0.096	0.236	0.252	0.307	0.384
rs6788895	RISK	0.538	0.157	0.396	0.030	0.193
rs743554	RISK	0.244	0.115	0.017	0.156	0.153
rs12355840	RISK	0.727	0.267	0.087	0.159	0.208
rs4808616	RISK	0.215	0.154	0.002	0.261	0.115
rs6569648	RISK	0.011	0.124	0.053	0.224	0.125
rs66823261	RISK	0.352	0.216	0.204	0.204	0.252
rs17350191	RISK	0.025	0.284	0.198	0.334	0.474
rs74911261	RISK	0.002	0.016	0.001	0.029	0.002
rs11076805	RISK	0.184	0.135	0.114	0.257	0.249
rs322144	RISK	0.184	0.379	0.319	0.565	0.337
rs113701136	RISK	0.122	0.258	0.220	0.281	0.233
rs77283072	RISK	0.336	0.392	0.105	0.155	0.252
rs10122055	RISK	0.582	0.473	0.782	0.279	0.272
rs4237533	RISK	0.306	0.488	0.276	0.554	0.390
rs10736303	RISK	0.082	0.494	0.430	0.474	0.499
rs1373925	RISK	0.254	0.517	0.549	0.404	0.589
rs7116745	RISK	0.573	0.324	0.146	0.188	0.101
rs7940177	RISK	0.019	0.229	0.212	0.092	0.168

rs8037816	RISK	0.116	0.640	0.560	0.622	0.560
rs12906805	RISK	0.067	0.597	0.366	0.501	0.409
rs4843437	RISK	0.305	0.373	0.097	0.428	0.320
rs2059287	RISK	0.518	0.300	0.279	0.187	0.291
rs2051591	RISK	0.510	0.414	0.109	0.415	0.497
rs5941428	RISK	0.296	0.571	0.533	0.608	0.421
rs13074711	RISK	0.388	0.095	0.142	0.114	0.047
rs3834129	RISK	0.556	0.412	0.211	0.475	0.241
rs3754934	RISK	0.260	0.055	0.198	0.048	0.290
rs12990906	RISK	0.129	0.499	0.477	0.572	0.322
rs10931936	RISK	0.189	0.434	0.304	0.283	0.138
rs3817578	RISK	0.217	0.052	0.198	0.047	0.290
rs10931936	RISK	0.189	0.434	0.304	0.283	0.138
rs1045485	RISK	0.050	0.076	0.001	0.120	0.024
rs2800691	RISK	0.384	0.291	0.356	0.242	0.280
rs4415084	RISK	0.350	0.442	0.453	0.591	0.515
rs7716600	RISK	0.170	0.298	0.482	0.204	0.261
rs12493607	RISK	0.104	0.275	0.706	0.337	0.377
rs4784227	RISK	0.042	0.313	0.255	0.254	0.222
rs2046210	RISK	0.663	0.272	0.362	0.321	0.318

rs11747675	RISK	0.170	0.246	0.270	0.390	0.347
rs4238807	RISK	0.179	0.406	0.454	0.265	0.231
rs12919689	RISK	0.607	0.346	0.540	0.228	0.470
rs73169113	RISK	0.014	0.095	0.193	0.189	0.219
rs10764446	RISK	0.082	0.197	0.016	0.326	0.265
rs4896144	RISK	0.008	0.056	0.055	0.073	0.123
rs4405256	RISK	0.056	0.297	0.375	0.268	0.467
rs59162058	RISK	0.011	0.016	0.029	0.031	0.053
rs1657248	RISK	0.021	0.154	0.145	0.132	0.095
rs150796751	RISK	0.008	0.075	0.010	0.086	0.148
rs3754934	MORTALITY	0.260	0.055	0.198	0.048	0.290
rs67918676	MORTALITY	0.120	0.084	0.001	0.134	0.046
rs145963877	MORTALITY	0.005	0.049	0.001	0.130	0.038
rs4717568	MORTALITY	0.133	0.550	0.666	0.609	0.586
rs1917618	MORTALITY	0.128	0.549	0.665	0.609	0.588
rs1546774	MORTALITY	0.124	0.549	0.665	0.610	0.587
rs1546773	MORTALITY	0.124	0.549	0.665	0.609	0.586
rs3803662	MORTALITY	0.567	0.390	0.619	0.291	0.273
rs188287402	TOXICITY	0.133	0.305	0.194	0.067	0.273
rs12657177	TOXICITY	0.089	0.085	0.222	0.066	0.095

rs75912034	TOXICITY	0.005	0.078	0.211	0.068	0.095
